# Annotation extension through protein family annotation coherence metrics

**DOI:** 10.3389/fgene.2013.00201

**Published:** 2013-10-11

**Authors:** Hugo P. Bastos, Luka A. Clarke, Francisco M. Couto

**Affiliations:** ^1^LaSIGE, Department of Informatics, Faculdade de Ciências, Universidade de LisboaLisboa, Portugal; ^2^Department of Chemistry and Biochemistry, BioFIG - Centre for Biodiversity, Functional and Integrative Genomics, Faculdade de Ciências, Universidade de LisboaLisboa, Portugal

**Keywords:** functional annotation, annotation extension, protein annotation coherence, annotation metrics, gene ontology

## Abstract

Protein functional annotation consists in associating proteins with textual descriptors elucidating their biological roles. The bulk of annotation is done via automated procedures that ultimately rely on annotation transfer. Despite a large number of existing protein annotation procedures the ever growing protein space is never completely annotated. One of the facets of annotation incompleteness derives from annotation uncertainty. Often when protein function cannot be predicted with enough specificity it is instead conservatively annotated with more generic terms. In a scenario of protein families or functionally related (or even dissimilar) sets this leads to a more difficult task of using annotations to compare the extent of functional relatedness among all family or set members. However, we postulate that identifying sub-sets of functionally coherent proteins annotated at a very specific level, can help the annotation extension of other incompletely annotated proteins within the same family or functionally related set. As an example we analyse the status of annotation of a set of CAZy families belonging to the Polysaccharide Lyase class. We show that through the use of visualization methods and semantic similarity based metrics it is possible to identify families and respective annotation terms within them that are suitable for possible annotation extension. Based on our analysis we then propose a semi-automatic methodology leading to the extension of single annotation terms within these partially annotated protein sets or families.

## 1. Introduction

The continuous development of high-throughput methodologies for biological molecule sequencing has led to an increase in the amount of raw biological data in need of further processing. The sequencing of a new biological molecule is normally followed by a functional annotation process that aims to provide functional descriptors elucidating its biological role. Functional annotations can be derived from either experimental determination or prediction. Generically, given supporting evidence, functional descriptors are assigned (with varying degrees of confidence) to their corresponding biomolecules. In fact, a functional annotation can be represented as the pair of a biomolecule (identifier) and corresponding functional descriptor.

Among biomolecules, proteins are of particular interest given their participation in practically every process occurring within living cells. Their functions can range from structural or mechanical support to the catalysis of vital metabolic biochemical reactions. Furthermore, their functional specification is very broad and can range from descriptors on general participation in biological processes, such as responses to oxidative stress, up to more specific descriptors, such as catalysis of particular biochemical reactions. It would be desirable to determine protein function via accurate and comprehensive chemical characterizations, if possible by experimental assessment, however, this process is expensive and time consuming. Instead, the most commonplace approach is the use of any of the several function prediction methodologies, relying on techniques ranging from sequence homology detection to text mining of the scientific literature. Most of these methodologies also rely heavily on computational power and can range from partial to full automation, thus enabling them to handle the barrage of biological sequence data currently being made available.

Proteins are commonly grouped into evolutionarily related groups known as *protein families*. Within a family each protein shares homology with all the other proteins, i.e., it descends from a common ancestor and usually retains significant sequence similarity. In turn that often (but not always) translates into similar three-dimensional structures and functions. Although sequence similarity alone is not sufficient to conclude protein homology, it nevertheless provides a reasonable cornerstone for many sequence alignment methods. Similarly, homology also does not guarantee functional similarity among proteins but provides a good starting point and is commonly used in several functional annotation methods. Hence, it is typically advantageous to group proteins into homologous families because of the potentially shared functions.

The emergence of biological ontologies and most notably the Gene Ontology (GO) (Ashburner et al., 2000) has greatly benefited the annotation efforts by providing a structured and controlled vocabulary of terms for the description of gene products. This standardization of human-readable functional descriptors also enables machine-readability thus being particularly useful in automated procedures. This in turn leads to an ever increasing availability and quality of protein annotations. The increasing popularity of GO terms for protein annotation has also led to the development of several associated semantic similarity based metrics that compare proteins based on their functions instead of their sequence or structure. GO semantic similarity can then be defined as the closeness in meaning between two terms or two sets of terms annotating two proteins. Under the assumption that when functional descriptors of two proteins are similar so are their functions, semantic similarity is then also referred to as *functional similarity*. However, caution must be exerted during the interpretation of annotation similarities since there are still issues that GO inherently does not solve, for instance, annotation bias and annotation incompleteness.

The functional descriptions of GO, given its ongoing and asymmetric growth, span a range of specificities (Alterovitz et al., 2010). Coupled with that, protein prediction methods assign either more specific or more generic annotation terms depending on the uncertainty level of the predictions being made. When comparing proteins annotated at different levels of completeness low semantic similarity values may then be reported. Therefore, the metrics used either have to account for these issues or adequate care must be taken when interpreting results.

The development of functional similarity metrics able to explicitly gauge the state of annotation incompleteness within a set of functionally related proteins is much required. We further postulate that by implementing these kind of metrics, we can identify functionally coherent sub-sets of proteins with a greater degree of annotation “completeness.” Using these identified sub-sets as specific function knowledgebases we can potentiate the annotation extension of the remaining members in a functionally related set that is still incompletely annotated, ultimately leading to a greater degree of annotation completeness for a given functionally related protein set.

## 2. Theory

### 2.1. Gene ontology

The GO consortium provides a structured and controlled vocabulary for the description of molecular phenomena in which proteins (and or gene products) are involved. Within each GO aspect the biological phenomena are described at different levels, thus this vocabulary is divided into three orthogonal ontology aspects that describe gene products in terms of their associated biological processes, cellular components and molecular functions (Ashburner et al., 2000). The *biological process* aspect of GO describes activities of sets of proteins interacting and involved in cellular processes, such as metabolism or signal transduction. The cellular localizations (such as the Golgi complex or the ribosome), where these processes take place are described by the *cellular component* aspect of the ontology. On the other hand, each protein can, independent of the surrounding environment, perform catalytic or binding elementary molecular activities thus being described by the *molecular function* aspect of the ontology. Structurally each ontology aspect is organized as a Directed Acyclic Graph (DAG), where each node represents a term and edges represent a relationship between those terms. Each term is identified by an alphanumeric code (e.g., GO:0001170) and its textual descriptors, including its name, definition, and synonyms if available. Currently, the existing relationships between GO terms can be of three types: *is_a*, *part_of* and *regulates*. While *is_a* and *part_of* relations are only established within each individual ontology aspect, *regulates* relations can occur across aspects.

**Figure 1:**
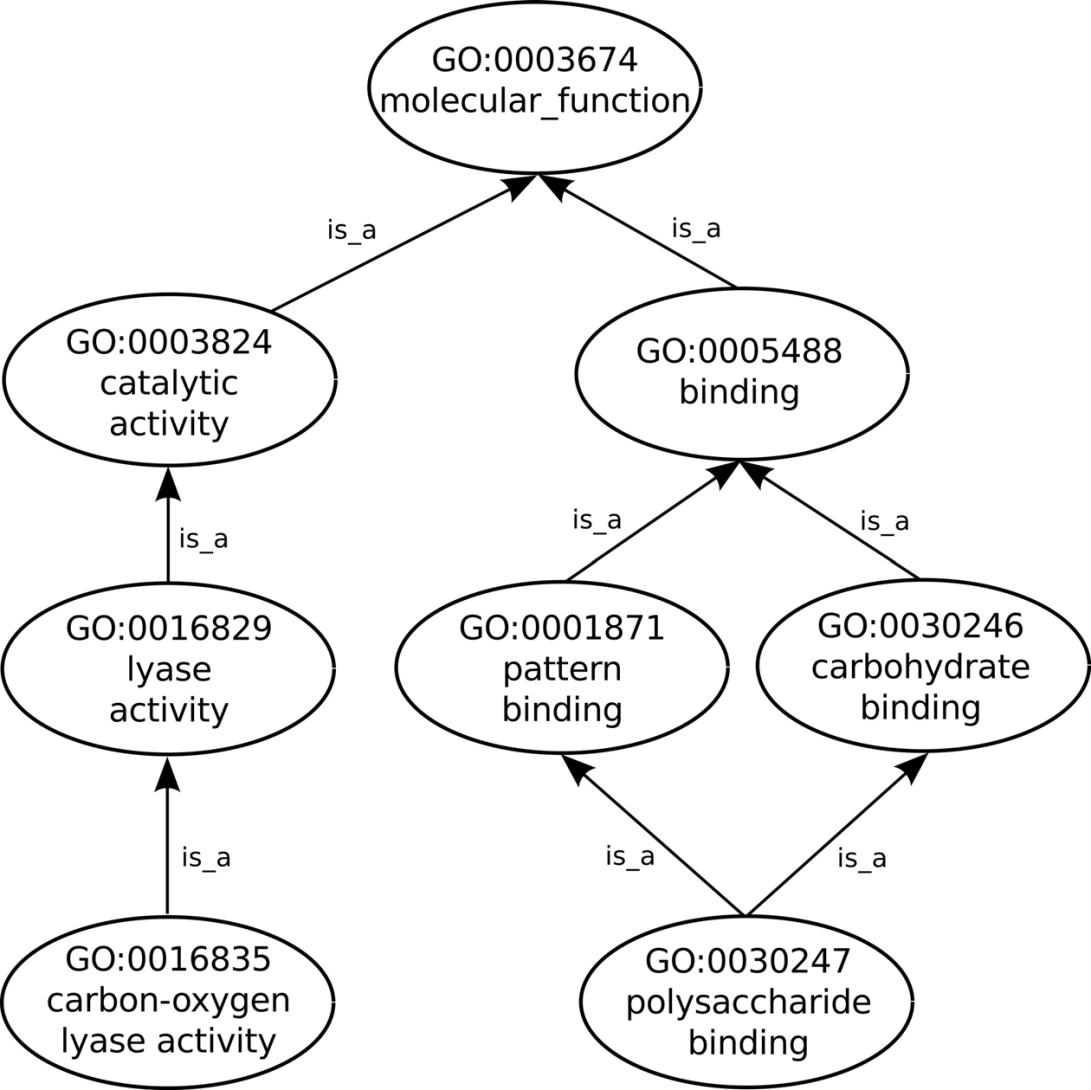
**Sub-graph of the GO *molecular function* ontology aspect depicting only *is_a* relationships**.

Proteins and other gene products are not actually part of GO which includes only terms that describe them. Nevertheless, the GO Consortium, via the Gene Ontology Annotation (GOA) project (Barrell et al., 2009), does provide annotations, such as previously defined as being the associations between gene products and the GO terms that functionally describe them. In order to fully describe a protein function any number of GO terms can be used to annotate the protein. Additionally, GO follows the true path rule which states that “the pathway from a child term all the way up to its top-level parent(s) must always be true”, thus as can be seen in Figure 1 any protein annotated to the term *polysaccharide binding* is also automatically annotated to its two parent terms: *carbohydrate binding* and *pattern binding*. In turn these two sibling terms are children of the term *binding*, a direct child of the root term *molecular_function*. Furthermore, each annotation linking a GO term to a protein is given an evidence code (ECO), which is an acronym identifying the type of evidence that supports that annotation, e.g., the IDA code (Inferred by Direct Assay) is assigned to annotations that are supported by that type of experiment.

### 2.2. Protein go annotation

Functional annotation is an essential step in the path of providing proteins their biological contexts and therefore facilitates knowledge exchange within the scientific community. Several methodologies exist for protein annotation but generally they can be divided into three major approaches: manual annotation (or curation), automatic annotation and the hybrid approach, semi-automatic annotation. Despite manual annotations produced by expert curators typically being of an higher quality level, this annotation approach does not scale up to the output of the high-throughput sequencing projects. Therefore, the bulk of protein annotations are produced via automated procedures. These typically rely on methods for transferring annotation terms from previously annotated protein sources to other unannotated (or incompletely annotated) proteins.

Using a controlled vocabulary like GO for protein annotation instead of free-text annotation solves several issues mostly common to many early annotation systems. Among those issues are the lack of annotation interoperability due to researcher subjectivity, the lack of vocabulary uniformity and problems arising from different scopes in function definitions. The scope of annotation can range from gene identification, cellular component specification and description of molecular interactions up to regulatory interactions between components of whole biological systems. This could present itself as an issue during the annotation process but is dealt with by the GO structure, where these scopes are divided into three orthogonal ontology aspects: *cellular component*, *molecular function*, and *biological process*. However, GO does not solve all annotation issues and even introduces new ones. The GO ontology aspects themselves are a product of mostly manual curation and their growth is linked to research bias, thus some parts of the ontology are more developed (have terms for more specific functions) than others (Pesquita and Couto, 2012). This is a source of incompleteness for annotation by limiting the maximum functional specificity that can be attributed to proteins. However, despite the availability of specific terms some annotation methodologies (mostly automatic) are unable to use them to annotate proteins with an high degree of confidence. Hence, this leads to a similar type of annotation incompleteness. On the other hand, a conservative annotation behavior may be desirable in order to mitigate possible annotation error propagation.

Typically, the automatic protein annotation systems do not actually produce *de novo* functional annotation terms. Instead, these systems commonly rely on methods for transferring annotation terms from previously annotated protein sources to other unannotated (or incompletely annotated) proteins. Thus, the typical workflow of an automatic annotation system includes a first stage where potential functional peers are identified. A second stage then involves the actual annotation transfer where functional terms are extracted from the functional peers and associated to the previously incompletely annotated or unannotated proteins.

The automatic procedures used for protein annotation can be divided into sequence-based approaches and structure-based approaches. Although three dimensional structure of proteins is generally more conserved than its sequence, the wider availability of sequence data over structural data allows for potentially greater annotation coverage with the former. Still, proteins with similar sequences typically possess evolutionary proximity, and to some extent, function conservation thus providing good approximations. In a similar sense, structure-based approaches can also compare protein structures in order to obtain similarity scores, but further details on structure-based approaches are out of the scope of this topic (see more at Sleator and Walsh, 2010).

**Figure 2:**
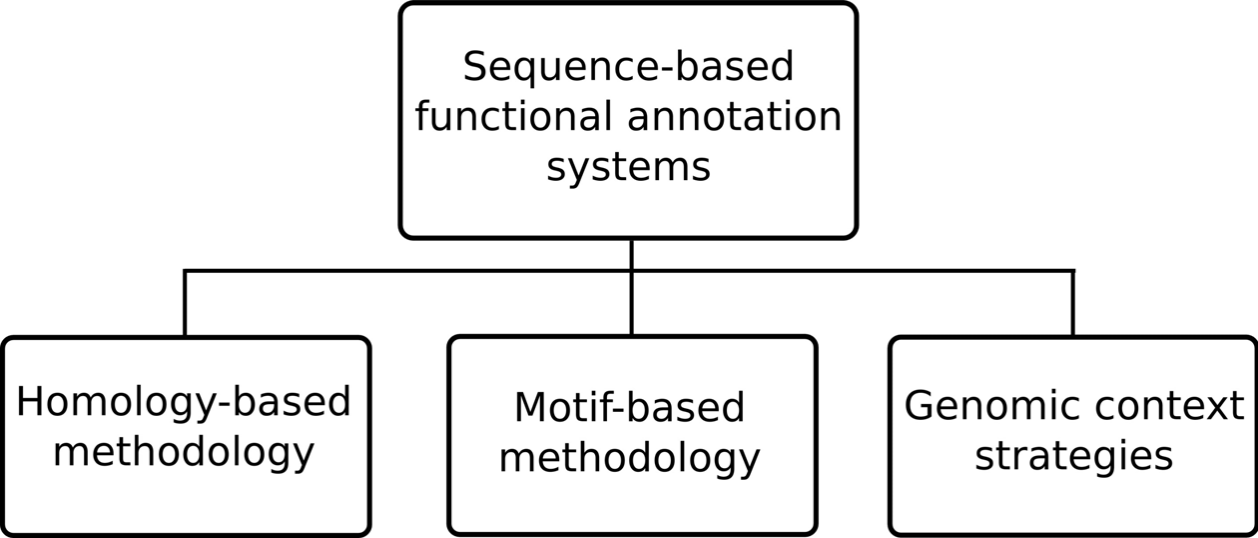
**Common approaches in sequence-based functional annotation systems**.

The sequence-based approaches can still be further sub-divided, as depicted in Figure 2, into three specific methodology types: homology-based, motif-based and genomic context strategies. Among the existing functional annotation systems the homology-based methodology is perhaps the most prevalent methodology. This type of methodology generally makes use of sequence alignment algorithms, such as the ubiquitous BLAST (Altschul et al., 1997), to compare unannotated query proteins against annotated sequences in a database. The underlying assumption is that similar sequences are most likely to have evolved from a common ancestor and thus retained similar functions. However, high sequence similarity does not always mean functional similarity (Rost, 2002) so annotation systems also employ additional techniques to handle known caveats. An example of a system using this approach is Blast2GO (Götz et al., 2008) where homologous sequences are retrieved from significant BLAST results under a given expectation value (*e*-value) threshold. In order to handle the possibility of annotation of short sequence matches with low *e*-values filtering by minimal alignment length (hsp-length) is allowed. An alternative to querying unannotated sequences against databases of annotated sequences, is to query them instead against known recurring patterns of motifs known to be associated with particular functions. This is the so-called sequence motif-based methodology where an annotation system uses either the patterns, rules and profiles of PROSITE (Sigrist et al., 2010), the fingerprints in PRINTS (Attwood, 2003), the family profiles from ProDom (Bru et al., 2005), the Hidden Markov Models (HMMs) from Pfam databases (Finn et al., 2010) or any other sequence motif type in order to perform functional inference.

Other alternative annotation strategies can be categorized under the denomination of genomic context strategies. These strategies subsume the gene neighborhood, gene clustering, Rosetta stone and phylogenetic profiles methods, which operate by identifying pairs of non-homologous proteins that co-evolve. Evolutionary pressure originates pairs of proteins that functionally collaborate and that: (i) are coded nearby in multiple genomes, (the gene neighborhood method); (ii) are components of an operon in prokaryotes, (the gene cluster method); (iii) can be fused into a single protein in some organisms, (the Rosetta stone method); (iv) are regularly both present or both absent within genomes, (the phylogenetic profiles method) (Bowers et al., 2004). Protein-protein interactions and gene expression data from microarray experiments have also been used as part of the functional peers identification methodology in some annotation systems. These genomic context methods can be used on annotation systems either individually or conjointly. (Overbeek et al., 1999) apply the gene clustering method on their system to infer functional coupling in prokaryotic genomes. (Zheng et al., 2002) also uses a clustering method but applied on phylogenetic profiles. Using microarray mouse expression data for nearly 40,000 known and predicted mRNAs in 55 mouse tissues (Zhang et al., 2004) were able to show that quantitative transcriptional co-expression is a powerful predictor of gene function. On the other hand, the Prolinks (Bowers et al., 2004) database uses the four genomic methods described above in combination to infer functional linkage between proteins through the identification of co-evolved pairs of non-homologous proteins. Similarly Phydbac (Enault et al., 2005), a gene function predictor system specialized in bacterial genomes also uses genomic context strategies in its workflow. Protein associations are generated by a combination of the phylogenetic profiles, the gene cluster and Rosetta stone methods. Both (Deng et al., 2002) and (Letovsky and Kasif, 2003) employ the theory of Markov random fields to infer a protein's functions using protein-protein interaction data and the functional annotations of a protein's interaction partners. (Chua et al., 2006) also developed a method for predicting protein function based on protein-protein interaction data, the difference being that in this case transitive relations are also considered for the predictions.

Prediction and assignment of protein function is seldom done in a deterministic way. While some general functions can be assigned deterministically to sequences, as protein function specificity rises the uncertainty of predicting an exact assignment does also. Thus, following the identification of functional peers it is common for annotation systems to employ an additional stage where term selection and transfer occurs. A confidence measure is usually associated with these term transfers, which often derives directly from probabilistic features from either statistical or machine learning methods employed for term selection, or alternatively, arbitrary empirical confidence measures from rule-based term selection methods. The methodologies used at the annotation transfer stage can be roughly grouped into three types: rule-based transfer, statistical transfer and machine learning transfer. One example of a rule-based methodology for annotation transfer occurs in the previously mentioned Blast2GO annotation system. There, for each candidate GO term, the highest similarity weighted by their ECO is considered. In addition the level of abstraction is also considered through the use of a rule counting the total number of GO terms unified at a given node weighted by a user set factor that controls the possibility and strength of abstraction. In the end, the annotation rule will only transfer the lowest terms in each branch that surpass an user defined threshold (Götz et al., 2008). A statistical-based annotation transfer methodology is used for example on the GOtcha (Martin et al., 2004) annotation system. GOtcha calculates probabilities for each term and its set of ancestors which allows some functions for a given sequence to be assigned with more confidence than others. Those probabilities are derived from two scores based on the expectation scores of pairwise matches between query sequences and database sequences and also the annotation distribution within each aspect of the GO ontology. On the other hand, the GOPET (Vinayagam et al., 2006) annotation system uses yet another type of approach: machine learning. In this system, GO terms associated to the retrieved homolog sequences are used in conjunction with several elaborate attributes, including sequence similarity measures, such as *e*-value, bit-score, identity, coverage score, and alignment length. Further attributes use GO-term frequency, GO term relationships between homolog sequences, the level of annotation within the GO hierarchy and homolog annotation quality which is calculated based on the ECO provided by the gene association tables of the GO mapped sequence databases. These attributes are used as training instances for support vector machines (SVM) which are then used to assign GO term annotation to the previously unannotated sequences.

### 2.3. Semantic similarity

In the context of ontology, semantic similarity can be defined as the closeness in meaning between two ontology terms or two sets of terms annotating two entities represented by a given metric. Typically, the semantic similarity between two proteins annotated with GO terms is also called functional similarity, since it presents a measure of how similar the protein functions are.

Semantic similarity measures for comparing terms in an ontology typically rely on two main approaches: edge-based and node-based. Edge-based approaches in their most simple form rely on counting the number of edges between two terms on the ontology graph, which conveys a distance measure that can easily be converted to a similarity measure (Rada et al., 1989). Thus, the shorter the distance between two terms, the more similar they are. Different edges can have different associated semantic values leading to more sophisticated metrics. On the other hand node-based approaches can be better suited for ontologies such as GO, where nodes and edges are not uniformly distributed. A commonly used node property is the information content (IC), which is a frequency-based measure of how specific a term is within a given corpus (Resnik, 1995). Conveniently, the GOA project provides a suitable body of GO annotations that can used as a corpus. The *IC* of term can then be given by Equation 1.

(1) $$IC(t)=-{\text{log}}_{2}f(t)$$

In Equation 1 *f*(*t*) is the probability of annotation of term *t*. Consequently, terms annotating many proteins will score a low *IC*, while specific terms annotating only a few proteins will score an high *IC*. Additionally, the *IC* values can be normalized in order to provide a more intuitive meaning.

GO-based semantic similarity for proteins is given by the comparison of the sets of GO terms annotating each protein being compared within each GO ontology aspect. Two main approaches, pairwise and groupwise (Pesquita et al., 2009) are typically used for this purpose. Pairwise approaches use semantic similarities between the GO terms annotating each protein, the semantic similarities are calculated for all possible pairs of terms between each set. Common among these approaches are variations such as the all pairs technique, where every pairwise combination is considered or the best pairs technique where only the best-matching pair for each term is considered. Global functional similarity scores between the actual proteins are usually obtained by averaging, summing or selecting the maximum of the pairwise similarity scores. For more on ontology-based semantic similarity check reviews by (Pesquita et al., 2009) and (Gan et al., 2013).

Several assessment studies have employed the developed semantic similarity measures for GO terms. There is no best measure for comparing terms, proteins or other gene products, it always depends on which specific task they are being used for. (Lord et al., 2003) were among the first to assess the performance of different semantic similarity measures in the context of GO. For that purpose they adapted and tested three measures: Resnik's (Resnik, 1995), Lin's (Lin, 1998), and Jiang and Conrath's (Jiang and Conrath, 1997) that were originally developed for the WordNet (Miller, 1995) taxonomy, a lexical database for the English language. These adapted measures were tested against sequence similarity using the average combination approach. Later, (Pesquita et al. (2008) also tested several measures against sequence similarity and found simGIC to provide overall better results. In contrast, (Guo et al., 2006) found simUI to be the weakest measure when evaluated for its ability to characterize human regulatory pathways, while it was found to perform fairly well when evaluated against sequence similarity in the assessment by Pesquita et al. (2008).

### 2.4. Term enrichment

Among the analysis operations involving GO terms, term enrichment analysis is one the most commonly used. Micro-array experiments often output lists which can represent hundred or thousands of genes found to be differentially regulated for a given condition under study. The purpose of term enrichment analysis is then to abstract from the individual genes and focus instead on a representative set of activity terms that summarize the particular biological activity differential, characteristic of the condition being studied. Those differentials (typically enrichment, although it can also be depletion) can be quantitatively measured resorting to commonly used statistical tests for this effect, such as the Fisher exact test, the Chi-squared test, the Hypergeometric distribution and Binomial distribution.

(Huang et al., 2009) collected and reviewed 68 bioinformatic enrichment tools categorizing them into three different classes, singular enrichment analysis (SEA), gene set enrichment analysis (GSEA) and modular enrichment analysis (MEA). Common to these three categories is the computation of *p*-values which for SEA is done for each term in a list of pre-selected genes deemed of interest, whereas GSEA needs no pre-selection and has experimental values integrated directly into *p*-value calculation. On the other hand MEA is similar to SEA but additionally factors term-term and gene-gene relations into the *p*-value calculations.

However, and despite the number of available enrichment tools there are still several unaddressed issues, even if we disregard issues stemming from experimental design and execution. These originate from variations in the sizes of the lists of genes, dependencies among genes or terms, annotation incompleteness and overall heterogeneity regarding specificity of annotation. And while the MEA methods try to address and even take advantage of the possible dependencies between genes or terms, issues pertaining to heterogeneous term availability or annotation distribution can still cause several problems and are still not optimally addressed.

## 3. Discussion

### 3.1. Case study

Consider, as case-study, the CAZy database (www.cazy.org) that describes the families of structurally-related catalytic and carbohydrate-binding modules (or functional domains) of enzymes that degrade, modify, or create glycosidic bonds (Cantarel et al., 2009). Its maintenance is done by a small team of curators that uses semi-automatic methods to keep it up-to-date. Even with part of the procedure being automatic there is still a large workload of manual curation that has to be performed by the specialized curators. Recently, the CAZy database has shifted from a schema where function was attributed to the complete enzyme sequence to a schema where function may be assigned just to the segment of the sequence involved in each function, the functional module. So far the CAZy families have been functionally annotated with Enzyme Commission (EC) numbers (Webb and NC-ICBMB, 1992). The EC number is a numerical classification for enzymes, based on the reactions they catalyze. The module-centric organization schema of the database can be complemented in such a way that functions, enzymatic or not, may be directly assigned to a specific segment of a sequence. In summary, CAZy is a curated knowledgebase of functionally related protein (module) families and despite not making use of GO as primary annotation system it still requires annotations with high specificity in order to achieve better characterization. Therefore, the CAZy families are good candidates for performing annotation coherence assessments and annotation extension studies.

The Polysaccharide Lyases (PL) are a group of enzymes that cleave uronic acid-containing polysaccharide chains via a β-elimination mechanism to generate an unsaturated hexenuronic acid residue and a new reducing end. Within the CAZy database these enzymes are classified into families and subfamilies based on amino acid sequence similarities, intended to reflect their structural features (Lombard et al., 2010). A quick assessment of the GO annotation status of these PL families was done using two simple naive metrics, GOscore and GOoccurence (Bastos et al., 2011) described by Equation 2 and Equation 3, respectively.

(2) $$GOscore(\text{fam})={\text{MAX}}_{\text{term }\in \text{fam}}\left[{\text{freq}}_{\text{fam}}(\text{term})\text{x}IC(\text{term})\right]\;\;\;\;$$

(3) $$GOoccurence(\text{fam})={\text{AVG}}_{\text{term }\in \text{fam}}\left[{\text{freq}}_{\text{fam}(\text{term})}\right]$$

Fundamentally, the GOscore metric is an indicator of the maximum *IC* expressed by the annotations of a family as conveyed through the most predominant and most informative term annotating a given family. On the other hand, the GOoccurrence metric expresses annotation coherence by averaging the frequency of all terms annotating one family. Hence, a family will report maximum functional annotation coherence (GOoccurrence = 1) when all terms are shared by all proteins in a given family. It should be noted that when applying this metric to sets of families of multifunctional proteins misinterpretations can be made if the multiple functions are not evenly shared and annotated within the protein set or family being measured.

### 3.2. Results

The incompleteness of annotation over a given protein space may lead to erroneous interpretations regarding functional coherence of that space. As mentioned previously we applied two annotation metrics, GOscore and GOoccurence to the PL families of the CAZy database. The results for both metrics are shown in Table 1 with the respective number of annotated proteins for each family. Upon inspection of the obtained GOoccurrence values, the families PL5, PL15, PL16, PL17, PL20 stand out as being the perfectly coherent families in terms of annotation (GOoccurrence = 1). Further and closer inspection of the actual annotation distribution within those families reveals that families PL5, PL16, PL17 are functionally mono-specific. This means that, for each of those families, there is only a single and common (known) molecular function activity performed by their proteins. Additionally, the reported GOscores for these families are also fairly high and thus indicate that they are annotated with functionally specific terms. Regarding families PL15 and PL20 they present deceptively high GOoccurrence values but these can be dismissed on account of the low number of annotated proteins (3 and 1, respectively) in those families. Given their low statistical support these two families are unsuitable for further analysis. Moreover, the only functional annotation in these two families is the *lyase activity* term. Considering their low *IC* (0.202) the functional information provided by these families is therefore also of little informative value.

**Table 1:** **GO annotation scores (GOscore and GOoccurrence) and respective size in number of annotated proteins for each CAZy family in the PL enzyme class**.

**Family**	**PL1**	**PL2**	**PL3**	**PL4**	**PL5**	**PL6**	**PL7**	**PL8**	**PL9**	**PL10**	**PL11**	**PL12**	**PL13**	**PL14**	**PL15**	**PL16**	**PL17**	**PL18**	**PL20**	**PL22**
Size	391	34	228	43	37	21	63	184	89	77	44	19	5	9	3	22	30	3	1	29
GOocc	0.146	0.798	0.306	0.373	1.000	0.405	0.288	0.303	0.128	0.261	0.325	0.586	0.550	0.420	1.000	1.000	1.000	0.667	1.000	0.880
GOscore	0.196	0.511	0.593	0.309	0.599	0.192	0.202	0.508	0.166	0.202	0.129	0.202	0.718	0.180	0.202	0.640	0.599	0.202	0.202	0.577

In turn, family PL22, despite appearing to be mono-specific, nonetheless has a GOoccurrence score of 0.880. This is in fact due to the penalization inflicted by 7 out of 29 proteins annotated proteins not being annotated with the most specific term *oligogalacturonide lyase activity*. Instead those 7 proteins are only annotated with *lyase activity*, an ancestor term of *oligogalacturonide lyase activity*. So, in this family, despite being mono-specific, it provides a clear case of annotation incompleteness that could lead to misinterpretations if we were to rely on coherence metrics alone. On the other hand, these annotations could be potentially extended to the *oligogalacturonide lyase activity* term. For instance, using all the proteins annotated to this term to create multiple sequence alignments, and subsequently creating position-specific scoring matrices, hidden Markov models or others statistical models these could be used to find matches on the 7 incompletly annotated proteins.

Another example, the PL3 family, despite having a similar GOscore (0.593), conversely has a rather low GOoccurrence score (0.306). In addition, by looking at the distribution of annotation terms within this family we can discover that all of its 228 proteins are annotated to the *pectate lyase activity* term. However, this otherwise coherent annotation is broken by 6 additional terms that annotate the family heterogeneously to a much lesser extent (only up to 2 proteins per term). Thus, here can be seen that the multi-functional nature of proteins can greatly affect the GOoccurrence metric. However, given the context of the PL enzyme class in which the PL3 family is inserted, if we were only to consider annotation terms that are children of *lyase activity* then we would obtain a considerable GOoccurrence improvement to a score of 0.798 (data not shown) for this particular family. The annotation terms that would be discarded, in this case, are clearly the product of secondary functional modules in the proteins that do not contribute to the global functional characterization of the family. Hence, their removal when accounting for family functional coherence is appropriate for this particular case. Regardless of any analitical assertion over their biological value, their low annotation count does not lend additional statistical support. That can be further confirmed through the use of enrichment analysis on this family and using the Benjamini-Yekutieli (Benjamini and Yekutieli, 2001) method, for an α = 0.01 only *pectate lyase activity* and *pectin lyase activity* are considered significant (corrected *p*-values of 0 and 9.8 × 10^−4^, respectively).

**Figure 3:**
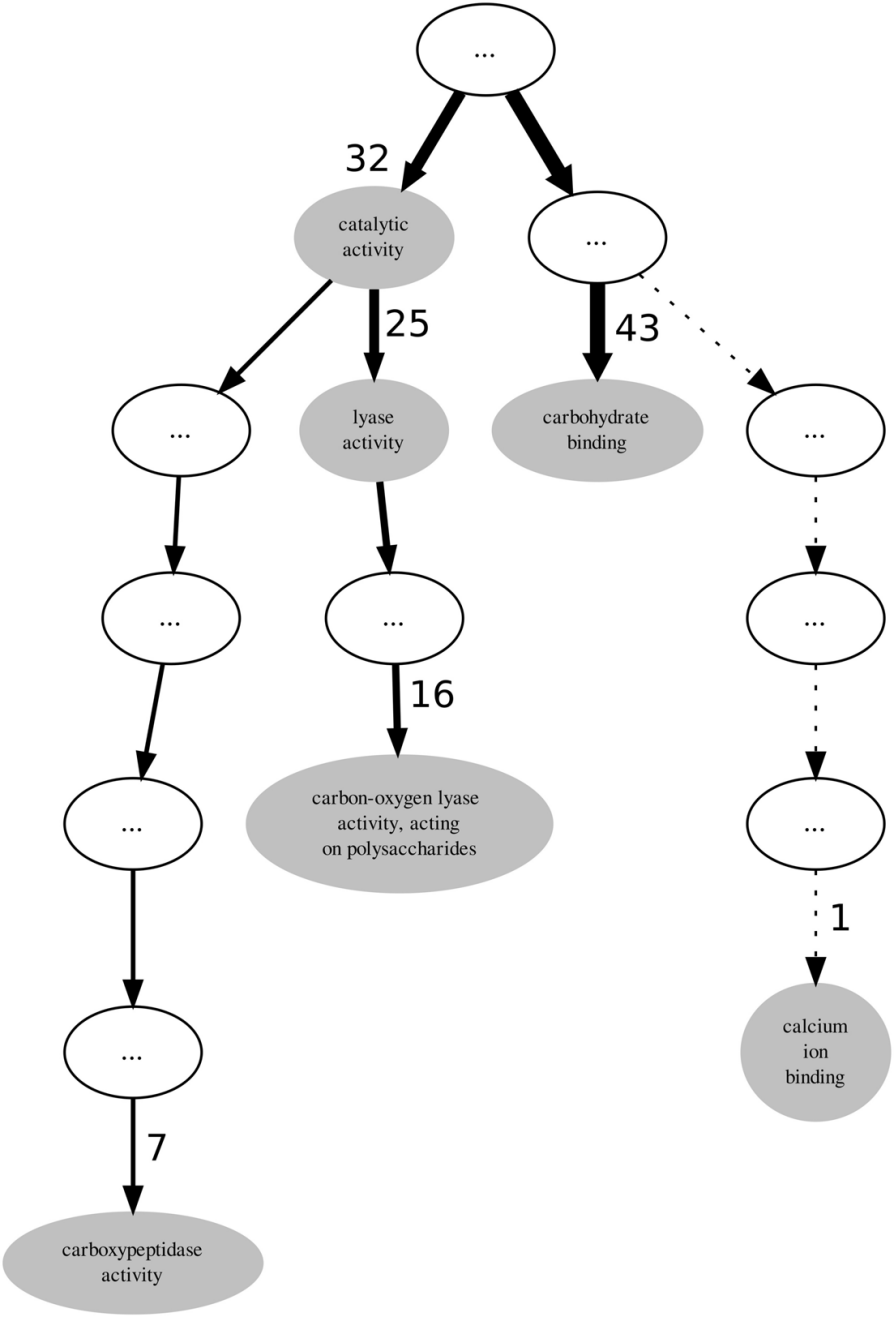
**Graph subsuming the GO molecular function aspect annotation of CAZy's PL4 family**.

Visualization can be very helpful when analysing GO term annotations for families or sets of proteins, thus we also used it in our analysis. PL4 is a moderately annotated family in terms of incompleteness which presents low values both for the GOscore and GOoccurrence metrics. The graph represented in Figure 3 subsumes the GO term annotations from the molecular function aspect in the PL4 family. The top unlabelled node on the graph is actually the root term *molecular_function* to which all the 43 sequences are annotated. It is important to notice that in the graph all indirect or inherited annotations are represented by unlabelled white nodes while direct annotations are represented by gray GO term-named nodes. It should also be noted that the direction of edges on the depicted graph in Figure 3 is reversed in relation to the actual GO graph. The edges in a typical GO graph represent the hierarchical *is_a* relations that hold between the molecular function aspect terms, and edge direction points from the outer leaf terms converging into a common root node, making the foundations of the true path rule that states that “the pathway from a child term all the way up to its top-level parent(s) must always be true” (Ashburner et al., 2000). On the other hand, the graph edges on Figure 3 actually represent the flow of proteins from the most generic root term into the more specific leaf GO terms. Additionally, edge thickness is proportional to the number of proteins “flowing down” from a parent node to a child node, and hence receiving a more specific annotation. Thus, these modified edges are particularly useful in providing visual cues regarding annotation specificity, homogeneity and functional relevance for a given protein family.

Again, given that the PL4 family belongs to the PL enzyme class it would be expected that all proteins within the family might be annotated to the *lyase activity* term. However, out of 43 proteins only 25 are annotated with the *lyase activity* term thereby leaving 18 proteins that potentially could also be annotated with it. By following the descendants of the *lyase activity* term down the graph we find that the term *carbon-oxygen lyase activity, acting on polysaccharides* annotates only 16 sequences. It is not unexpected that the number of annotated protein decreases as we walk down an annotation graph toward the leaf terms. Given that the bulk of annotation is performed by automatic methods it becomes more difficult to provide protein annotations at more functionally specific levels with enough confidence. However, for the PL4 family the most specific GO term is *carboxypeptidase activity* annotating 7 proteins. Although this term is not a descendent of the *lyase activity* term, 6 of the proteins annotated with it are also annotated with with *carbon-oxygen lyase activity, acting on polysaccharides* term. Unlike the PL3 family, for the case of the PL4 family it is not as simple to resolve the multi-functional nature of their proteins and just excluding terms that are not descendants of *lyase activity* is not an obvious option.

InterPro (Zdobnov and Apweiler, 2001) is a resource that can be used to scan protein sequences against an extensive collection of signatures from multiple and diverse databases, and allows the presence of domains and important sites useful to be predicted for protein functional analysis. Therefore, by using the InterProScan on the PL4 family sequences we can obtain the resulting matches against the InterPro signatures. A quick visual comparison of the signature profiles of both *lyase activity* annotated proteins and non-*lyase activity* annotated proteins leads us to infer that the latter can in fact also be annotated to the *lyase activity* term with reasonable confidence given the similarity of the signature profiles. However, as can be seen in Table 2, despite the term *lyase activity* being statistically significant, for α = 0.01 and a Benjamini-Yekutieli corrected *p*-value, the *IC* (normalized for the GOA annotation corpus) is relatively low, therefore indicative of a differentially low informative value. According to the term enrichment corrected *p*-values, the term *carbohydrate binding* has the greater statistical significance (among all the direct annotations in family PL4). However, intuitively it can be seen that this term, despite being biologically relevant, does not provide a great information increment, since it has the third lowest *IC* value in Table 2. The term *carbon-oxygen lyase activity, acting on polysaccharides* ranks second in terms of significance but its *IC* is also only slightly higher than the one for *carbohydrate binding*. It is actually the third ranked term for statistical significance, *carboxypeptidase activity* that has the greatest *IC* even though it is not even a descendant of *lyase activity*. Both *calcium ion binding* and *catalytic activity* fall below the previously chosen threshold of significance. The former can be explained by the fact that it has only one annotation occurrence, and is most likely not relevant for the PL4 family functional profile. The lack of significance of the latter term is explained by its ubiquitousness both within the CAZy families and the GOA annotation corpus which in turn also reflects itself as a low IC.

**Table 2:** **GO term enrichment for CAZy family PL4 with Benjamini-Yekuteli corrected *p*-values, normalized *IC* and number of annotations**.

**GO term**	***p*-value (corr)**	**IC (norm)**	**Annotations**
Carbohydrate binding	1.87e-47	0.658	43
Carbon-oxygen lyase activity, acting on polysaccharides	7.50e-23	0.699	16
Carboxypeptidase activity	3.43e-12	0.813	7
Lyase activity	1.76e-10	0.404	25
Calcium ion binding	4.75e-01	1.000	1
Catalytic activity	1.00e+00	0.166	32

**Figure 4:**
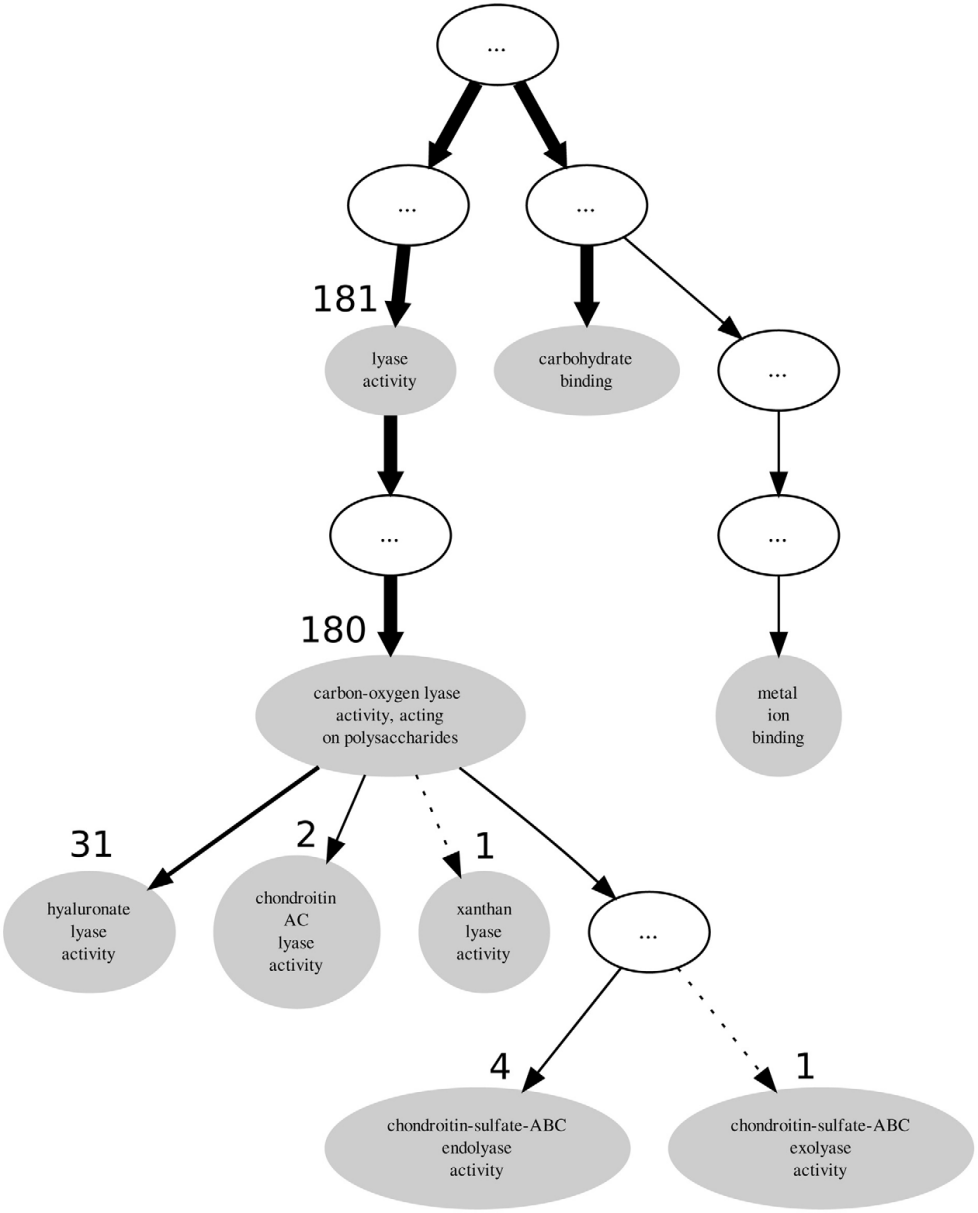
**Graph subsuming the GO molecular function aspect annotation of CAZy's PL8 family**.

Descendant terms of *lyase activity* can also lead to a reduced annotation coherence, as measured simplistically by the GOoccurrence metric. Any non-uniform annotation distribution within a family will penalize this metric. For the PL8 family, as can be seen in Figure 4, the penalization comes in part from the multiple descendants of the *lyase activity* term. There are five leaf-terms that are descendants of *lyase activity* in family PL8, but presenting an asymmetrical distribution regarding the number of proteins they annotate. As show in Table 3 all of these five terms are enriched in family PL8, however only the term *hyaluronate lyase activity* annotates sufficient proteins to potentially create a support corpus that would allow annotation extension for this term and within this family. Hence, there are still 149 candidate proteins annotated with the *carbon-oxygen lyase activity, acting on polysaccharides* term that can be asserted for extension with the *hyaluronate lyase activity term*. As for the remaining sibling terms they can not be dismissed as irrelevant for the family characterization, and are part of this family set of relevant activities but lowering the value of the GOocccurrence metric.

**Table 3:** **GO term enrichment for CAZy family PL8 with Benjamini-Yekuteli corrected *p*-values, normalized *IC* and number of annotations**.

**GO term**	***p*-value (corr)**	**IC (norm)**	**Annotations**
Carbon-oxygen lyase activity, acting on polysaccharides	8.15e-306	0.699	180
Carbohydrate binding	1.18e-186	0.658	178
Hyaluronate lyase activity	2.00e-095	1.000	31
Chondroitin-sulfate-ABC endolyase activity	3.69e-011	1.000	4
Chondroitin AC lyase activity	1.24e-005	1.000	2
Chondroitin-sulfate-ABC exolyase activity	5.49e-003	1.000	1
Xanthan lyase activity	5.49e-003	1.000	1
Lyase activity	3.37e-002	0.404	181
Metal ion binding	1.00e+00	0.687	2

### 3.3. Proposed approach

**Figure 5:**
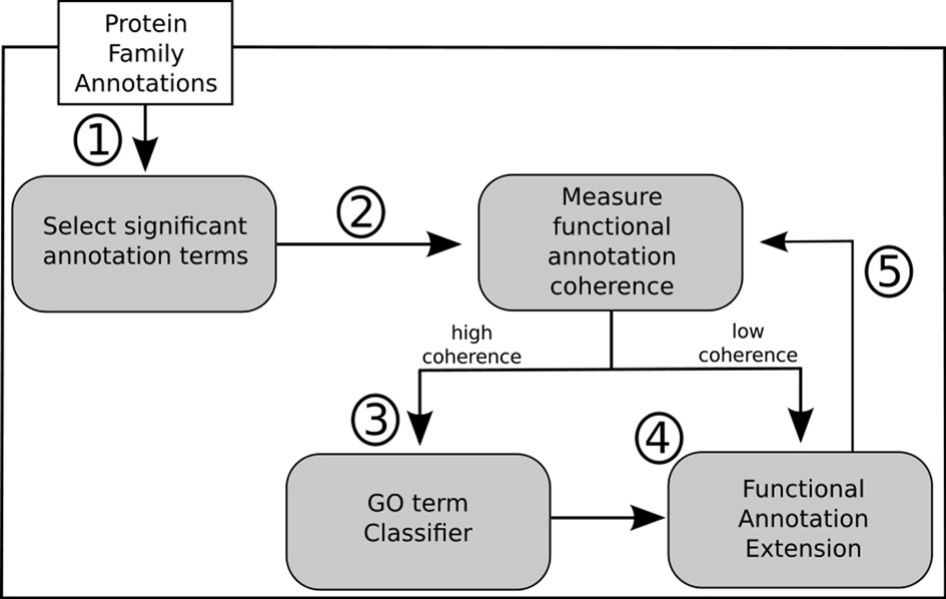
**Outline of proposed methodology for annotation extension**.

In light of the results discussed above we propose a general methodology for extending GO annotations in protein families as depicted in Figure 5. Consider a set of protein families created by curators within a given biological knowledge domain. A certain level of functional similarity is inherently expected from these families. Following an initial collection of terms annotating each of these families a statistical enrichment can then ensue. The commonly used technique of statistical enrichment allows the filtering out of possible annotation terms that are not characteristic of a family. At this point (Step 1) additional manually created rules might be beneficial in order to capture not only statistical support but potentially biological meaning related to the specific context domain of the protein families. Following the process of selecting the relevant term annotations for a given family, functional annotation coherence in a family can be asserted through the use of groupwise semantic similarity metrics (Step 2). A protein family showing greater annotation coherence may supply sub-sets of protein (sequences) that can be used to create multiple sequence alignments. These can subsequently be used to create position-specific scoring matrices, hidden Markov models or other statistical models that can be used for classification. Also, any other available or obtainable protein feature from a sub-set of proteins sharing an annotation can theoretically be used with several machine learning techniques in order create individual GO term classifiers. Visualization methods can be helpful in making this procedure semi-automatic. Following that course of action subsuming annotation graphs, like the ones in Figures 3, 4, can be dynamically generated. These annotation graphs can also be made interactive in order to allow navigation through the individual nodes. Hence, considering that each node represents an annotation term, the graph can then be linked with the sub-set of proteins annotated by that term in a given family. This allows the selection of proteins which will contribute with features (sequences or otherwise) to construct the single GO term classifiers (Step 3). In turn, these classifiers can then be used for the purpose of extending functional annotation on incompletely annotated proteins within the given protein family (Step 4). By submitting the families to the annotation metrics the coherence differential can be gauged after each iteration of annotation extension (Step 5). It should be noted that the overall family coherence metrics used should be selected or customized in order to take into account the particular knowledge domain being assessed. Of particular notice is that extensions are done per annotation term, and each protein (and family) can have multiple functions and thus terms associated to them.

## 4. Conclusions

Ideally, proteins should be annotated in a way that fully describes their functional activities. However, even within the boundaries of current knowledge, this is seldom the case. As we try to compare protein sets, such as families, based on their functional annotations this heterogeneity of functional annotation becomes a greater issue. Annotation incompleteness in annotations can lead to false interpretations about the existing functional inter-similarity within a given protein set (or family). In order to avoid erroneous interpretations on heterogeneous protein sets or families (in terms of annotation specificity), functional comparisons are usually done at conservative levels. This means that by comparing families at conservative annotation levels we would also be comparing terms with lower *IC* and hence obtaining less informative conclusions.

Resources such as the CAZy database provide high-quality classifications of segments of the protein space into functionally related families. These kind of protein families present themselves as an opportunity and a knowledgebase from which we can benefit in order to provide annotation extension methodologies. Considering that any given protein family is a functionally related set of sequences, then the heterogeneity of annotation specificity can be explored within each family. Thus, sub-sets of homogeneous annotation in a family can be used to produce classifiers which can potentially extend other proteins within the same family that are under-annotated. This proposed methodology should be regarded as a generic approach guided at mitigating some of the current issues with annotation incompleteness, and despite not being suitable for all annotation incompleteness states it should allow for an increased extension of annotation over the ever increasing protein space. It should be particularly useful when applied in tandem with protein families from databases like CAZy where proteins despite being grouped together into functionally close families they still do not focus on functional annotation. It should be noted that the coherence metrics presented here are only to illustrate typical annotation baseline patterns and are not intended to be used in fully automated procedures or to address issues like the measuring of coherence in sets of multifunctional proteins on their own. However, customized metrics derived from groupwise semantic similarity measures can be implemented for each specific knowledge domain under study in order to automate most of the procedure in the suggested methodology.

## Acknowledgments and funding

Portuguese Fundacão para a Ciência e Tecnologia (www.fct.pt/) through the financial support of the SPNet project (PTDC/EIA-EIA/119119/2010), the SOMER project (PTDC/EIA-EIA/119119/2010) and the PhD Grant ref. SFRH/BD/48035/2008 and through the LASIGE multi-annual support.

### Conflict of interest statement

The authors declare that the research was conducted in the absence of any commercial or financial relationships that could be construed as a potential conflict of interest.
